# Genetic diversity and population structure in *Bactrocera correcta* (Diptera: Tephritidae) inferred from mtDNA *cox1* and microsatellite markers

**DOI:** 10.1038/srep38476

**Published:** 2016-12-08

**Authors:** Yu-Jia Qin, Nopparat Buahom, Matthew N. Krosch, Yu Du, Yi Wu, Anna R. Malacrida, Yu-Liang Deng, Jia-Qi Liu, Xiao-Long Jiang, Zhi-Hong Li

**Affiliations:** 1 College of Plant Protection, China Agricultural University, Beijing 100193, China; 2 Office of Agriculture Regulation, Department of Agriculture, Ladyao, Chatuchak, Bangkok 10900, Thailand; 3 School of Earth, Environmental and Biological Sciences, Queensland University of Technology, G.P.O. Box 2434, Brisbane 4000, QLD, Australia; 4 Yunnan Entry-Exit Inspection and Quarantine Bureau, Kunming 650228, China; 5 Academy of State Administration of Grain, Beijing 100037, China; 6 Dipartimento di Biologia Animale, Università degli studi di Pavia, Piazza Botta, I27100 Pavia, Italy; 7 Xishuangbanna Entry-Exit Inspection and Quarantine Bureau, Jinghong 666100, China; 8 General Administration of Quality Supervision, Inspection and Quarantine of the People’s Republic of China, Beijing 100088, China

## Abstract

*Bactrocera correct*a is one of the most destructive pests of horticultural crops in tropical and subtropical regions. Despite the economic risk, the population genetics of this pest have remained relatively unexplored. This study explores population genetic structure and contemporary gene flow in *B. correcta* in Chinese Yunnan Province and attempts to place observed patterns within the broader geographical context of the species’ total range. Based on combined data from mtDNA *cox1* sequences and 12 microsatellite loci obtained from 793 individuals located in 7 countries, overall genetic structuring was low. The expansion history of this species, including likely human-mediated dispersal, may have played a role in shaping the observed weak structure. The study suggested a close relationship between Yunnan Province and adjacent countries, with evidence for Western and/or Southern Yunnan as the invasive origin of *B. correcta* within Yunnan Province. The information gleaned from this analysis of gene flow and population structure has broad implications for quarantine, trade and management of this pest, especially in China where it is expanding northward. Future studies should concentrate effort on sampling South Asian populations, which would enable better inferences of the ancestral location of *B. correcta* and its invasion history into and throughout Asia.

*Bactrocera correcta* (Bezzi) (Diptera: Tephritidae), the guava fruit fly, is one of the most destructive pests of many tropical and subtropical fruits and vegetables, such as guavas, mangoes, citruses, melons and chili peppers, causing production losses and quality degradation^1^,^2^. Considering that the species has a broad host range, is highly adaptable, and has a high reproductive ability and dispersal capacity^3^,^4^, it has been regulated as a quarantine pest by many countries, including China^1^,^5^. *Bactrocera correcta* was first reported in Bihar, India in 1916^6^, where it is thought to have originally diverged from its common ancestor^7^,^8^. At present, it is distributed throughout South and East Asia, from Bhutan in the west to China and Vietnam in the east^9^,^10^,^11^,^12^. The species has been recorded in the United States, where it was first detected in California in 1986, but has not yet established due to timely prevention and control measures^13^. In China, *B. correcta* was first discovered in the Yunnan Province (Yuanjiang and Mosha) in 1982^14^, and the infestation of this fly has become more entrenched in Yunnan in recent years^15^.

Yunnan is located in southwest China, adjacent to Myanmar, Laos and Vietnam, from which entry of exotic species into Yunnan has been facilitated by similar environmental conditions^4^. As such, Yunnan is considered to represent a transition zone for invasive fruit flies^16^, where the native ranges of some species blur into a northward-moving invasion front. One example of this is *B. correcta* (Bezzi), which first entered China through Yunnan^17^ and has since moved from Yuanjiang to Yuanmou in Yunnan province, and also to Panzhihua, Sichuan province^4^. Futhermore, ecological modelling data suggested that, at certain times of year, *B. correcta* is capable of establishment across the whole of China^18^. Given the notable rapid spread of *B. correcta* in China, its economic importance, and the risk of this species being introduced, establishing and invading other regions of China argue for greater understanding of population relationships and invasion routes.

Information regarding genetic diversity, genetic structure and gene flow are key issues when developing management strategies^19^,^20^. The mitochondrial (mtDNA) cytochrome oxidase subunit I (*cox1*) gene is easily amplified^21^, maternally inherited and relatively fast-evolving, which allows derivation of recent female-specific evolutionary histories. In contrast, microsatellites (SSRs) are nuclear, bi-parentally inherited, highly polymorphic and easy to isolate, making them particularly informative in the study of contemporary biological invasions^16^,^22^. Thus, these two sets of molecular markers are extensively used to study the population genetic structure of insect pests^20^,^23^,^24^, including *Bactrocera* fruit fly species^16^,^25^,^26^,^27^.

Here, we assess population genetic structure within Yunnan Province via dense geographical sampling to determine patterns of gene flow and dispersal within China. We then place these patterns in a broader geographical context by sampling other locations across the species’ distribution. In doing so, we build on previous localized evidence concerning the genetic structure and invasion history of *B. correcta* (e.g., within Thailand)^28^, and attempt to infer the route by which this species entered China. Specifically, we test whether *B. correcta* is expanding northward in China via gradual movement from adjacent populations or if new, adventive northern populations are derived from further away. Likewise, we seek to determine whether any locations within Yunnan Province show signs of having been the original invasive location in China and from which the invasion progressed. Additionally, we incorporate limited public data from South Asia (India, Sri Lanka) to address hypotheses concerning this region as the putative ancestral location of the species. Taken together, this study provides foundational data for understanding the population dynamics and genetic structure of *B. correcta*, which will contribute greatly toward development of control measures for this fly in Yunnan.

## Methods

### Sample collection, DNA extraction, *cox1* sequencing and microsatellite genotyping

**Figure 1:**
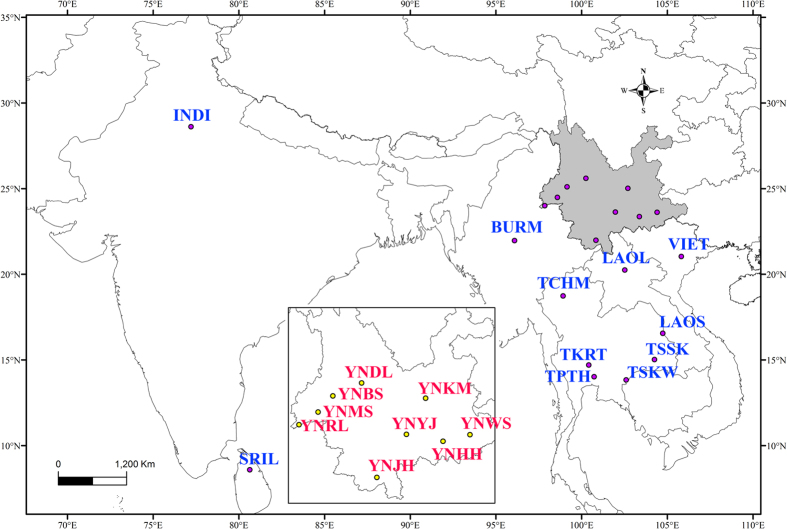
Geographical location of 20 sampled sites across South and Southeast Asia. Note: Insert figure: Yunnan Province, China. The map was created in ArcGIS 10.2 software (ESRI Inc., Redlands, CA, USA). URL http://www.esri.com/software/arcgis/arcgis-for-desktop.

Specimens of *B. correcta* were collected from 18 sites in China (Yunnan), Laos, Myanmar, Thailand, and Vietnam from 2008 to 2013^29^ using methyl eugenol (ME) traps (Table 1 and Fig. 1)^30^. All flies were stored in 100% ethanol at −20 °C prior to DNA extraction. In addition to new data collected here, we added 12 *cox1* sequences from GenBank for flies from India (accession numbers GU323781 and GU323782) and Sri Lanka (accession numbers JQ692856, JQ692753, JQ692641, JQ692784, JQ692787, JQ692756, JQ692631, JQ692832, JQ692711 and JQ692676)^31^.

Genomic DNA was extracted from each specimen using the Tissue/Cell DNA Mini Kit (Tiangen Biotech, Beijing, China). A 658 bp *cox1* fragment was amplified and sequenced according to the method of Liu *et al*.^32^. Both directions of the *cox1* sequence from each individual were assembled using DNAMAN 5.2 (Lynnon Corporation, Quebec, Canada). To delete low-quality sections, all sequences were aligned with the standard sequences of *B. correcta* from BOLD using Clustal X^33^ to generate 600-bp *cox1* sequences. Unique sequences were deposited in GenBank with accession numbers KU669296–KU670076. Information about the twelve microsatellite loci and amplifying methods analyzed here^34^. Fluorescently labeled fragments were detected on an ABI PRISM 377 Genetic Analyzer, with ROX-500 size standard (Microread, Beijing, China). Allele size was analyzed by GeneScan V3.7 program (Applied Biosystems, Beijing, China).

### Marker summary statistics and intra-population genetic diversity

For *cox1* data, the nucleotide composition and variable positions were visualized using MEGA 6^35^. The nucleotide diversity (*π*), haplotype diversity (*Hd*) and number of haplotypes for each geographical population of *B. correcta* were estimated using DNASP 5.1^36^.

For microsatellite data, the number of alleles (*N*_A_), number of effective alleles (*N*_E_), observed heterozygosity (*H*_O_), expected heterozygosity (*H*_E_) were calculated each population using POPGENE 1.32^37^. Allelic richness (AR) and gene diversity (HS) were calculated using FSTAT 2.9.3.2^38^. Frequency of null allele (AN) was estimated using GENEPOP 4.1^39^. The same software was also used to check for genotypic linkage disequilibrium and for departure from Hardy-Weinberg Equilibrium (HWE) after sequential Bonferroni correction.

### Population genetic structure

Pairwise *F*_ST_ was calculated for both types of markers using Arlequin 3.5 to measure the degree of genetic differentiation between pairs of populations and corrected for the effect of multiple tests by using a modification of the false discovery rate method^40^. Isolation-By-Distance (IBD) was examined by testing the correlation between *F*_ST_/(1-*F*_ST_)^41^ ln-scaled geographical distances using Mantel tests^42^ in Arlequin 3.5 with 1000 permutations. Google^TM^ Earth 5.2^43^ was used to determine the linear geographical distances between each pair of sampling sites of *B. correcta*, the correlation analysis results were plotted in SPSS v16.0 (SPSS Inc., Chicago, IL).

Evolutionary relationships among *cox1* haplotypes were inferred using a haplotype network, constructed under the median-joining (MJ) method in NETWORK 4.6^44^. Bayesian clustering of individuals based on microsatellite genotypes was performed in STRUCTURE 2.0^45^ to infer genetic structure among the 18 studied populations of *B. correcta*. We set the number of clusters (K) from 1 to 10 and conducted 10 independent runs for each value of K. Each run consisted of a burn-in period of 50,000 steps, followed by 100,000 Markov chain Monte Carlo (MCMC) repetitions with a model allowing admixture. ΔK values^46^ were computed to select the most likely number of K using the online resource Structure Harvester^47^ that explained the structure in data. We then conducted model to summarize cluster membership coefficient matrices for each value of K with CLUMPP 1.1.2^48^, and plotted using DISTRUCT 1.1^49^. To identify the optimal number of groups (K) for sequences, spatial analysis of molecular variance was performed using SAMOVA 1.0^50^ taking into account the longitude and latitude information. The supported groups K was selected with the highest *F*ct value associated with the subdivision scheme by repeating the analysis with K ranging from 2 to 9. Hierarchical analysis of molecular variance (AMOVA) was performed using Arlequin 3.5 to evaluate the distribution of molecular variance among groups, populations and individuals for both types of markers based on the grouping strategy from STRUCTURE and SAMOVA.

### Demographic history

The demographic history analysis of all *cox1* sequences from 20 populations (including India and Sri Lanka) were examined using mismatch distribution and neutrality tests in Arlequin 3.5 with 1000 bootstrap replicates. Six parameters were calculated: effective population size before expansion (*θ*_**0**_), effective population size after expansion (*θ*_**1**_), Time of populations expansion (T), Tajima’s *D*, Fu’s *F*_S_ and sum of square deviation (SSD) between expected and observed mismatch distribution.

### Inter-population migration rate estimates

The GENECLASS v2.0 program^51^ was used to assign/exclude populations as donors or receivers of individuals on the basis of multilocus genotypes. For each individual in a population of *B. correcta*, this program computes the probability that it belongs only to that population, the probability of being a migrant from each of the other populations and the probability of being a migrant to other populations^16^,^25^. We used the standard criterion, which applies Bayesian statistics to calculate probabilities^52^, and Monte Carlo resampling method^53^, which calculates the accurate inclusion/exclusion critical values. We simulated 10,000 genotypes for each population with a threshold probability value of 0.01.

## Results

### Marker summary statistics and intra-population diversity

In total, we sampled 781 *B. correcta* from 18 sites across China and Southeast Asia. Inclusion of 12 additional *cox1* sequences from GenBank from India and Sri Lanka, produced a final alignment of 600 bp for 793 individuals. The A + T content was 63% (28.8% A and 34.2% T), higher than the G + C content (16.5% G and 20.5% C). This nucleotide composition is similar to *B. dorsalis*^26^ and *B. cucurbitae*^27^. Of the 600 nucleotide positions, 62 variable positions were observed (10.33%), including 19 singleton variable positions and 43 parsimony informative positions. Sixty-three haplotypes (designated H1-H63) were observed across the 20 populations of *B. correcta*. Of these, 30 haplotypes were shared by at least two populations (47.62%), with the most frequent haplotype H5 present in 17 populations. The number of haplotypes for per population ranged from 1 to 22 (Table 2). Three types of basic descriptive indices, namely haplotype diversity (Hd), nucleotide diversity (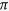) and average number of nucleotide differences (k) were calculated to measure genetic diversity within populations (Table 2). Polymorphisms were found in 19 populations (Hd = 0.898, on average). Among them, Western Yunnan (YNRL, YNMS and YNBS) and SRIL showed a higher diversity, YNHH in the eastern displayed a lowest Hd and lowest nucleotide diversity was found in Vietnam neighboring to YNHH. However, there was no signal of decreased diversity in more northern populations of China compared with southern or western populations.

The same 781 individuals of *B. correcta* that were sequenced for *cox1* were genotyped for 12 microsatellite loci. A total of 185 alleles were observed across 12 loci, ranging from 8 to 21 per locus (Supplementary Table S1). Consistent with the *cox1* data, YNMS from the western Yunnan presented the highest genetic diversity (Hs = 0.714) while VIET was the lowest (Hs = 0.444) followed by YNHH (Hs = 0.617) (Table 3).

### Population genetic structure

Genetic distance among populations estimated using pairwise *F*_ST_ values (Table 4) showed that, for *cox1*, YNHH and BURM were consistently significantly different to most other populations (ranging from 0.293 for YNRL/YNHH to 0.066 for YNJH/YNBS). Microsatellites by contrast suggested that most pairwise comparisons were significantly different; however, actual *F*_ST_ values were generally lower than 0.1, suggesting low population structure. An exception was the YNHH population, which was supported as more different to all other sites, with *F*_ST_ estimates ranging from 0.1 to 0.149, average 0.122. Mantel tests showed no significant relationship between genetic (*F*_ST_/(1-*F*_ST_)) and geographical distances. (*cox1* data: r^2^ = 0.011, P = 0.146; microsatellite data: r^2^ = 0.00018, P = 0.869), indicating the absence of IBD (Supplementary Fig. S1).

**Figure 2:**
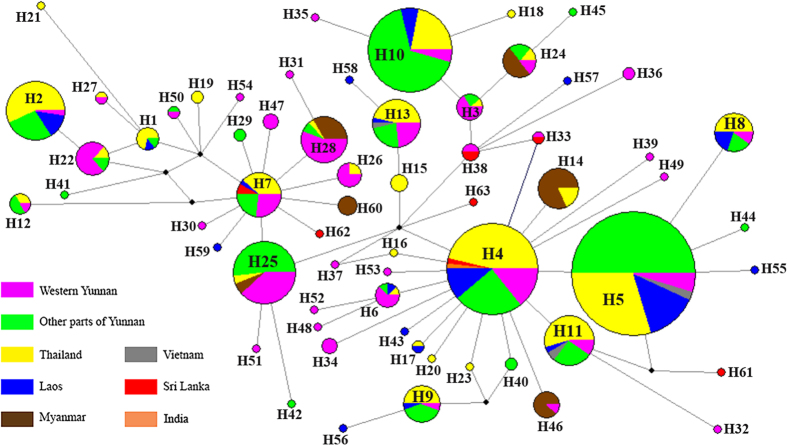
Median-Joining haplotype network of *B. correcta* based on mtDNA *cox1* data. Note: Size of nodes and pie segments were proportional to haplotype frequency; small black circles represent median vectors (roughly equivalent to hypothetical unsampled haplotypes); length of the branched is proportional to number of mutational changes between haplotypes.

The median-joining network constructed from 63 haplotypes demonstrated that several haplotypes were highly common and shared by many locations. In particular, H4 and H5, which were separated by a single mutation, were common across the sampled range and were connected to several low frequency tip haplotypes, implying that they may represent putative ancestral haplotypes (Fig. 2, Supplementary Fig. S2). Myanmar was the only location did not share either of these common haplotypes, but does share haplotypes with Yunnan and Thailand (H14, H24, H28, H46). Western Yunnan (Sites YNBS, YNMS and YNRL) displayed a high level of genetic variability in possessing 15 unique haplotypes, one of which is shared exclusively with Sri Lanka (H38).

**Figure 3:**
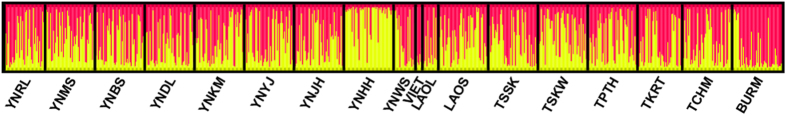
Bayesian results based on STRUCTURE among 18 populations of B.*correcta* at K = 2, individuals were grouped by collection site according to Table 1, each individual was represented by a vertical bar displaying membership coefficients.

Bayesian clustering analysis of microsatellite genotypes implemented in STRUCTURE showed that the maximum value for the estimated likelihood of K was found at K = 2 (Supplementary Fig. S3). Visualisation of cluster membership coefficients suggests that flies from the Site YNHH formed a single cluster separate to all other locations (Fig. 3). SAMOVA analysis also suggested the same grouping strategy, with the greatest proportion of variation among groups under a K = 2 hypothesis (*F*_CT_ = 0.12284) (Supplementary Table S2). AMOVA analyses performed according to these two groups (YNHH separated from all other populations) showed genetic differentiation among groups accounted for 12.34% and 7.46% for *cox1* and microsatellite data, respectively. All fixation indices, including *F*_CT_, *F*_SC_, *F*_ST_, *F*_IS_ and *F*_IT_ were highly significant (P < 0.01) (Supplementary Table S3).

### Demographic history

Neutrality tests performed on the total *cox1* dataset produced significant negative Tajima’s *D* and Fu’s *F*_S_ values (Table 2) and the mismatch distribution was unimodal (Supplementary Fig. S4), supporting a model of population expansion (P_SSD_ > 0.05). Ratios between estimated effective population size after expansion (*θ*_1_) and effective population size before expansion (*θ*_**0**_), which can serve as an estimate of the extent of population growth, indicated that *B. correcta* exhibited a certain degree of population growth in all the populations (Table 2).

### Inter-population migration rate estimates

Bi-directional migration rates among populations of *B. correcta*, estimated based on microsatellite data, show marked variation across the sampled geographical distribution, ranging from 0 (YNHH into YNMS) to 0.727 (TCHM into VIET). In these analyses, migration rate estimates below 0.100 imply restricted gene flow, whereas moderate to high gene flow is suggested by values greater than 0.100. Interestingly, the migration rates from the YNHH and VIET populations to other populations were all below 0.100, except for YNHH into VIET (m = 0.122). Meanwhile, estimated migration rates were also very low between BURM and other populations. The probability with which individuals were assigned to their own populations varied from 0.344 (YNWS) to 0.501 (BURM) (Table 5).

## Discussion

Understanding population structure and gene flow among regionsare very important aspects in the management of quarantine fruit flies. In this study, we obtained data from both mitochondrial and nuclear DNA markers of an extensive sampling of *B. correcta* in Yunnan Province, China, and neighboring countries. Our aim was to examine population structure and gene flow within Yunnan and to place Chinese diversity and structure in a regional context.

The clearest signal of population structure across the sampled locations supported the population YNHH, from eastern Yunnan, as being significantly structured from all other populations. This pattern was exemplified by low levels of estimated gene flow between this site and all others, and manifests in this site being supported as a separate genetic entity in Bayesian clustering analysis of microsatellite genotypes. Yunnan Province is characterized geologically as a longitudinal range-gorge region, where the mountain chains tend to run south to north, potentially blocking the spread of mobile insects eastward^4^,^54^. Genetic differentiation may thus be the result of natural barriers that limited gene flow from Eastern Yunnan westward and drove divergence of this population. Alternatively, this pattern may be representative of a separate origin for the YNHH population from a currently unsampled population, but presumably not from nearby.

Elsewhere across the sampled distribution, there were a small number of locations that were supported as significantly structured. Firstly, Myanmar, situated southwest of Yunnan and west of Southeast Asia also exhibited some evidence for significant genetic differentiation from other populations. This pattern may be associated with significant mountain ranges and/or reduced trade with other Asian countries that act to limit direct and indirect gene flow between Myanmar and surrounding regions. Myanmar displayed a close relationship with adjacent western Yunnan and Thailand, and high gene flow was inferred from these locations into Myanmar, in contrast to minimal migration in the opposite direction. This pattern has also been described in the highly invasive species *B. dorsalis*^16^,^25^, which is otherwise largely panmictic across much of Asia. Secondly, Vietnam is supported by microsatellite data as significantly different from most other sampled populations. Like Myanmar, there was reduced migration out of Vietnam to other locations, along with reduced diversity. It is unclear what might drive this pattern; however, a combination of trade practices and geographical barriers to dispersal may each play a role, or alternatively it could be driven by the small sample size for this population.

More broadly, resolved patterns are consistent with recent demographic expansion in this species. Significant negative Tajima’s D and Fu’s *Fs* indices, unimodle mismatch distributions and non-significant SSD values all supported the hypothesis of a sudden population expansion model. As *B. correcta* is a phytophagous and highly polyphagous fruit fly, presence of abundant hosts may aid its regional dispersal. Combined with highly frequent fruit trade between Yunnan and other Asian countries, this likely presents advantageous conditions for the long-distance dispersal of this fly^3^,^55^,^56^, which is considered to be on-going.

Within China, *B. correcta* was first recorded in southern Yunnan Province in 1982^14^, a region that represents one of the gateways into Southeast Asia with potential invasion via trade routes along the Makhong River or the Kunming-Bangkok international highway^57^,^58^. Our data provides no insight on the origins of the Chinese populations other than that they do not differ greatly in genetic profile from other locations across southeast Asia. Nevertheless, western Yunnan exhibited higher diversity than other Chinese populations. It is generally accepted that fruit flies invading new areas are most likely to first establish in regions that have abundant host plants and suitable climate^59^, characteristics that are exemplified in western Yunnan. Further, given that older populations often demonstrate higher levels of genetic diversity than more recently established populations^60^,^61^, we argue that our genetic data supports a scenario of *B. correcta* having established first in western Yunnan before expanding into other regions in China.

Patterns across the rest of Yunnan Province shed some light on movement among populations and the potential invasion history of *B. correcta* in the region. In particular, populations in southern and eastern Yunnan displayed lower genetic diversity than western populations. This suggests that these populations may be the result of gradual eastward migration from an initial invasion into western Yunnan. This is contrary to previous hypotheses that the region was an original entry point for *B. correcta* into China, which were based on its geographical proximity and horticultural trade relationships with neighbouring countries, along with the first detection of *B. correcta* in China occurring in southern Yunnan. Instead, this scenario finds little support in our data, although it cannot be conclusively excluded.

Our inclusion of Indian and Sri Lankan *cox1* sequence data allows some comment on the hypothesis that *Bactrocera* species may have originated in India^7^,^8^. We can propose the type of evidence that would be required to reject this hypothesis specifically, low genetic diversity and external/tip haplotypes in the *cox1* network for South Asian locations. In marked contrast: and despite low sample size, there was high haplotype diversity (7 haplotypes from 12 sequences across both sites), with these being mostly internal to the network and often shared between many other locations. Thus, these data provide some evidence for these populations being more ‘ancestral’ than Southeast Asian populations, indicatingthat we cannot reject the idea that *Bactrocera* originated in India. The limited data for India and Sri Lanka restricts our deductive capacity. Further sampling will be required to determine the geographical origin of *B. correcta* and resolve patterns of gene flow and dispersal between India and other Asian countries.

We applied two sets of molecular markers to avoid any bias due to the use of only a single marker. Mitochondrial DNA corresponds to the maternal lineage, making it sensitive to selective neutrality, loss of mutation-drift equilibrium and male-to-female sex ratio balance^62^, and is particularly informative for inferring phylogeographical patterns. Nuclear microsatellites, on the other hand, are biparentally inherited and evolve faster than mtDNA, making them more suitable for analysis of contemporary gene flow. Hence, each marker type provides resolution of patterns at slightly different evolutionary scales, and the limited observed discrepancies are likely to be due to the different evolutionary history of each marker. For example, many more pairwise comparisons of population structure (using *F*_ST_ indices) were significant for microsatellites than for *cox1*. We argue that this is most likely driven by the faster mutation rate of the former, although it may possibly represent some degree of female-biased dispersal.

In conclusion, we provide evidence based on combined independent molecular markers that there is high gene flow among most Southeast Asian populations of *B. correcta*, with exceptions in eastern Yunnan, Myanmar and Vietnam. We provide support for hypotheses of first entry of this species into China occurring in western Yunnan, with gradual dispersal eastward. Given current global warming trends, which will render more of northern China ecologically suitable for *B. correcta*^18^, and the notable rapid spread of this invasive species, we argue that this species warrants urgent attention to understand and manage this invasion front. Moreover, early detection and control measures need to be enhanced to avoid or slow the rate of new invasions in China.

## Additional Information

**How to cite this article**: Qin, Y.-J. *et al*. Genetic diversity and population structure in *Bactrocera correcta* (Diptera: Tephritidae) inferred from mtDNA *cox1* and microsatellite markers. *Sci. Rep.* **6**, 38476; doi: 10.1038/srep38476 (2016).

**Publisher’s note:** Springer Nature remains neutral with regard to jurisdictional claims in published maps and institutional affiliations.

## Supplementary Material

Supplementary Information

## Figures and Tables

**Table 1:** Sample information of the 20 populations of *B. correcta* used in this study.

Country	Collection site	Code	Sample size COI/SSR	Coordinates	Collection year
China (Yunnan Province)	Jinghong	YNJH	50/50	100°50′E 21°59′N	2008
	Honghe	YNHH	50/50	103°22′E 23°22′N	2011
	Wenshan	YNWS	22/22	104°24′E 23°37′N	2011
	Yuanjiang	YNYJ	50/50	101°58′E 23°38′N	2008
	Kunming	YNKM	50/50	102°42′E 25°01′N	2011
	Dali	YNDL	50/50	100°15′E 25°36′N	2011
	Baoshan (West)	YNBS	50/50	99°09′E 25°06′N	2012
	Mangshi (West)	YNMS	50/50	98°35′E 24°26′N	2008
	Ruili (West)	YNRL	41/41	97°51′E 24°00′N	2008
Thailand	Pathum Thani	TPTH	50/50	100°44′E 14°01′N	2011
	Nakorn Ratchasima	TKRT	45/45	101°25′E 14°42′N	2011
	Sakaew	TSKW	50/50	102°36′E 13°50′N	2011
	Sisaket	TSSK	50/50	104°15′E 15°01′N	2009
	Chiang Mai	TCHM	50/50	98°55′E 18°44′N	2010
Laos	Sawannaket	LAOS	50/50	104°44′E 16°33′N	2013
	Luang Phabang	LAOL	16/16	102°31′E 20°15′N	2009
Vietnam	Hanoi	VIET	7/7	105°49′E 21°02′N	2012
Myanmar	Mandalay	BURM	50/50	96°05′E 21°58′N	2011
India	New Delhi	INDI	2*/−	77°12′E 28°36′N	2009
Sri Lanka	Anuradhapura	SRIL	10*/−	80°38′E 8°35′N	2007

^*^Source from GenBank.

**Table 2:** Genetic diversity indices and demographic history parameters of *B. correcta* based on *cox1* data.

Population	Sample size	N	*Hd*	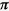	*k*	*θ*_0_	*θ*_1_	T	Tajima’s D	Fu’s *F*s	SSD
YNJH	50	15	0.834	0.00648	3.887	0.0123	5.2307	7.5606	−0.6731	−2.5423	0.0101
YNHH	50	4	0.549	0.00530	3.179	0.0018	2.4904	7.0801	1.6292	5.7939	**0.2849**
YNWS	22	9	0.896	0.00935	5.610	0.0070	15.5762	7.1523	−0.0975	0.6336	**0.0526**
YNYJ	50	9	0.812	0.00755	4.528	0.0018	7.1741	6.9922	−0.1096	2.1215	0.0725
YNKM	50	13	0.753	0.00584	3.506	0.0018	4.5923	6.8731	−0.8089	−1.6478	0.0539
YNDL	50	8	0.703	0.00514	3.084	0.0000	4.7073	5.8398	0.4472	1.1753	0.0686
YNBS	50	22	0.941	0.00774	4.644	0.0051	19.0210	5.4961	−1.2399	**−7.8074**	0.0113
YNMS	50	22	0.941	0.00822	4.935	0.9174	17.7515	4.9414	−1.0347	**−7.1837**	0.0023
YNRL	41	19	0.943	0.00727	4.361	0.0000	17.2192	5.4102	−1.0495	**−6.4774**	0.0051
TPTH	50	13	0.852	0.00748	4.491	0.0035	6.1316	9.5234	−0.4066	−0.5361	0.0156
TKRT	45	17	0.921	0.00806	4.838	0.1775	9.3005	6.9336	−0.8210	−3.2140	0.0173
TSKW	50	11	0.856	0.00894	5.364	0.0018	8.9453	8.8438	0.4624	1.4867	0.0269
TSSK	50	14	0.863	0.00719	4.311	1.6383	6.2317	6.0820	−0.6363	−1.3346	0.0144
TCHM	50	12	0.881	0.00598	3.585	1.7385	7.5732	2.7461	−0.7547	−0.8889	0.0066
LAOS	50	14	0.797	0.00600	3.602	0.0000	4.0503	8.4609	−1.0674	−2.2173	0.0138
LAOL	16	7	0.792	0.00897	5.383	0.0035	8.2617	10.6250	−0.0303	1.1771	**0.1244**
VIET	7	3	0.667	0.00317	1.905	0.0000	3.5428	3.9570	−0.3303	1.2221	0.1446
BURM	50	6	0.795	0.00705	4.232	0.0053	12.2113	6.7695	1.7143	4.7496	**0.0731**
INDI	2	1	0.000	0.00000	0.000	0.0000	0.0000	0.0000	0.0000	0.0000	**0.0000**
SRIL	10	7	0.933	0.00644	3.867	0.0000	22.5391	4.6719	−0.0250	−1.3725	0.0050
All	793					0.408	83.438	5.826	**−1.8710**	**−25.2909**	0.0022

N: number of haplotypes in each population; *Hd*: haplotype diversity;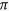: nucleotide diversity; *k*: average numbers of nucleotide differences; *θ*_0_: effective populations sizes before expansion; *θ*_1_: effective populations sizes after expansion; T: time of population expansion; SSD: sum of square deviation between expected and observed mismatch distribution under the sudden expansion model; bold values were significant at P < 0.05.

**Table 3:** Genetic variability in 18 populations of *B. correcta* based on microsatellite data.

Population	Sample size	*N*_A_	*N*_E_	*H*_O_	*H*_E_	*A*_R_	*A*_N_	*H*_S_
YNJH	50	9.583	3.560	0.579	0.681	4.719	0.070	0.682
YNHH	50	4.583	2.484	0.336	0.515	3.296	0.114	0.517
YNWS	22	5.583	2.868	0.374	0.597	4.089	0.130	0.602
YNYJ	50	8.750	3.394	0.642	0.693	4.686	0.038	0.693
YNKM	50	6.750	3.074	0.407	0.613	4.111	0.126	0.615
YNDL	50	6.083	2.801	0.423	0.603	4.006	0.108	0.605
YNBS	50	7.500	3.291	0.419	0.636	4.458	0.134	0.638
YNMS	50	8.417	3.804	0.633	0.713	4.881	0.062	0.714
YNRL	41	7.000	3.019	0.434	0.626	4.383	0.123	0.629
TPTH	50	7.583	3.117	0.480	0.629	4.339	0.101	0.631
TKRT	45	6.417	2.741	0.398	0.591	3.882	0.120	0.593
TSKW	50	7.333	3.123	0.525	0.638	4.393	0.072	0.639
TSSK	50	7.500	3.099	0.528	0.646	4.389	0.071	0.647
TCHM	50	7.917	3.369	0.498	0.656	4.501	0.095	0.658
LAOS	50	6.167	2.921	0.403	0.621	4.115	0.135	0.623
LAOL	16	4.833	2.926	0.381	0.615	4.026	0.138	0.623
VIET	7	2.833	2.033	0.464	0.445	2.833	0.027	0.444
BURM	50	5.167	2.741	0.437	0.572	3.754	0.094	0.574

*N*: sample size; *N*_A_: mean number of alleles; *N*_E_: mean number of effective alleles; *H*_O_: mean observed heterozygosity; *H*_E_: mean expected heterozygosity; *A*_R_: mean allelic richness; *A*_N_: mean frequency of null alleles; *H*_S_: gene diversity.

**Table 4:** Pairwise *F*_ST_ of *B. correcta* based on *cox1* data (below diagonal) and microsatellite data (above diagonal).

	YNJH	YNHH	YNWS	YNYJ	YNKM	YNDL	YNBS	YNMS	YNRL	TPTH	TKRT	TSKW	TSSK	TCHM	LAOS	LAOL	VIET	BURM	INDI	SRIL
YNJH	—	0.108	0.019	0.007	0.046	0.038	0.054	0.021	0.035	0.026	0.034	0.023	0.019	0.008	0.020	0.017	0.062	0.051		
YNHH	0.239	—	0.123	0.100	0.081	0.121	0.114	0.128	0.109	0.139	0.123	0.128	0.134	0.123	0.101	0.138	0.149	0.141		
YNWS	0.037	0.157	—	0.039	0.051	0.043	0.055	0.045	0.028	0.038	0.030	0.050	0.034	0.019	0.026	0.020	0.057	0.038		
YNYJ	0.061	0.059	0.015	—	0.054	0.046	0.050	0.012	0.041	0.024	0.045	0.029	0.020	0.014	0.025	0.036	0.078	0.073		
YNKM	0.070	0.083	0.065	0.010	—	0.042	0.021	0.059	0.019	0.051	0.023	0.083	0.052	0.046	0.020	0.030	0.075	0.056		
YNDL	0.180	0.258	0.059	0.127	0.180	—	0.028	0.050	0.022	0.046	0.026	0.059	0.046	0.035	0.018	0.021	0.043	0.042		
YNBS	0.066	0.236	−0.004	0.079	0.125	0.036	—	0.041	0.007	0.043	0.028	0.077	0.048	0.041	0.018	0.031	0.068	0.054		
YNMS	0.113	0.261	0.014	0.117	0.176	0.041	0.000	—	0.032	0.021	0.047	0.040	0.023	0.015	0.030	0.034	0.097	0.071		
YNRL	0.144	0.293	0.029	0.140	0.205	0.032	−0.002	−0.008	—	0.029	0.010	0.060	0.029	0.027	0.006	0.005	0.058	0.042		
TPTH	−0.009	0.184	0.001	0.023	0.045	0.134	0.043	0.087	0.112	—	0.024	0.051	0.001	0.002	0.022	0.021	0.069	0.066		
TKRT	0.014	0.208	−0.010	0.039	0.083	0.084	0.003	0.031	0.051	−0.003	—	0.065	0.022	0.019	0.010	0.006	0.053	0.034		
TSKW	0.031	0.141	−0.022	0.012	0.059	0.088	0.029	0.048	0.073	0.001	−0.002	—	0.049	0.034	0.053	0.045	0.094	0.077		
TSSK	−0.002	0.175	−0.005	0.016	0.039	0.115	0.032	0.074	0.097	−0.018	−0.007	−0.002	—	0.000	0.018	0.014	0.060	0.069		
TCHM	0.017	0.217	0.036	0.040	0.046	0.148	0.056	0.112	0.136	0.003	0.015	0.035	−0.001	—	0.013	0.015	0.058	0.051		
LAOS	0.010	0.169	0.047	0.023	0.010	0.181	0.092	0.150	0.181	0.001	0.038	0.036	0.001	0.005	—	0.005	0.061	0.050		
LAOL	0.001	0.164	−0.024	0.009	0.025	0.120	0.037	0.064	0.100	−0.020	−0.003	−0.023	−0.019	0.010	0.001	—	0.059	0.048		
VIET	0.031	0.249	0.085	0.042	0.034	0.277	0.128	0.202	0.234	0.018	0.059	0.067	0.025	0.012	−0.009	0.034	—	0.047		
BURM	0.126	0.241	0.058	0.108	0.149	0.102	0.047	0.066	0.073	0.097	0.064	0.087	0.081	0.084	0.132	0.101	0.183	—		
INDI	−0.159	0.176	−0.106	−0.122	−0.075	0.150	−0.057	0.011	0.068	−0.174	−0.154	−0.116	−0.178	−0.188	−0.177	−0.131	−0.040	−0.051	—	
SRIL	0.068	0.194	−0.016	0.033	0.098	0.043	−0.013	0.004	0.014	0.033	−0.005	0.010	0.018	0.062	0.083	0.030	0.165	0.040	−0.058	—

Bold values were significant after multiple tests correction.

**Table 5:** Migration rate (m) between population pairs of *B. correcta* calculated by GENECLASS 2.0 based on microsatellite data.

	YNJH	YNHH	YNWS	YNYJ	YNKM	YNDL	YNBS	YNMS	YNRL	TPTH	TKRT	TSKW	TSSK	TCHM	LAOS	LAOL	VIET	BURM
YNJH	0.419	**0**.**003**	0.12	0.362	**0**.**093**	**0**.**066**	0.123	0.313	0.144	0.218	0.149	0.21	0.237	0.319	0.119	0.128	**0**.**005**	**0**.**025**
YNHH	0.283	0.412	**0**.**099**	0.35	0.258	0.113	0.283	0.294	0.231	0.186	0.196	0.291	0.157	0.205	0.149	**0**.**06**	**0**.**013**	**0**.**078**
YNWS	0.515	**0**.**001**	0.344	0.411	0.246	0.139	0.273	0.348	0.268	0.302	0.228	0.234	0.295	0.39	0.239	0.217	**0**.**01**	**0**.**095**
YNYJ	0.362	**0**.**016**	**0**.**096**	0.441	0.102	**0**.**067**	0.102	0.318	0.144	0.253	0.156	0.206	0.259	0.255	0.11	0.123	**0**.**007**	**0**.**023**
YNKM	0.341	**0**.**025**	0.193	0.403	0.383	0.157	0.43	0.391	0.386	0.313	0.349	0.121	0.288	0.332	0.272	0.175	**0**.**009**	0.108
YNDL	0.472	**0**.**008**	0.16	0.437	0.219	0.401	0.382	0.472	0.416	0.346	0.34	0.306	0.359	0.415	0.275	0.2	**0**.**026**	0.152
YNBS	0.237	**0**.**002**	0.115	0.278	0.242	0.116	0.407	0.394	0.332	0.248	0.227	0.104	0.221	0.26	0.225	0.153	**0**.**007**	**0**.**085**
YNMS	0.225	**0**	**0**.**042**	0.261	**0**.**072**	**0**.**046**	0.17	0.39	0.15	0.157	0.103	0.113	0.164	0.203	**0**.**07**	**0**.**083**	**0**.**001**	**0**.**027**
YNRL	0.338	**0**.**013**	0.131	0.334	0.261	0.171	0.417	0.48	0.395	0.248	0.247	0.152	0.251	0.281	0.213	0.177	**0**.**014**	0.114
TPTH	0.425	**0**.**005**	0.106	0.446	0.178	0.174	0.286	0.427	0.257	0.451	0.277	0.2	0.417	0.483	0.254	0.222	**0**.**024**	**0**.**059**
TKRT	0.522	**0**.**013**	0.277	0.52	0.415	0.324	0.479	0.528	0.471	0.509	0.464	0.238	0.527	0.564	0.428	0.335	**0**.**027**	0.146
TSKW	0.362	**0**.**008**	0.102	0.342	0.055	**0**.**058**	**0**.**075**	0.268	0.107	0.204	0.147	0.434	0.227	0.268	**0**.**062**	0.136	**0**.**017**	**0**.**03**
TSSK	0.445	**0**.**002**	0.1	0.457	0.208	0.146	0.284	0.434	0.302	0.425	0.339	0.215	0.433	0.486	0.284	0.277	**0**.**01**	**0**.**052**
TCHM	0.473	**0**.**009**	0.124	0.408	0.142	0.117	0.219	0.402	0.2	0.395	0.252	0.221	0.385	0.415	0.244	0.21	**0**.**011**	**0**.**057**
LAOS	0.46	**0**.**013**	0.189	0.447	0.274	0.243	0.393	0.455	0.351	0.421	0.376	0.27	0.433	0.463	0.39	0.276	**0**.**028**	**0**.**092**
LAOL	0.508	**0**.**006**	0.266	0.388	0.205	0.16	0.378	0.439	0.333	0.433	0.394	0.281	0.47	0.531	0.361	0.356	**0**.**012**	0.108
VIET	0.724	0.122	0.501	0.672	0.597	0.422	0.559	0.481	0.614	0.686	0.451	0.455	0.496	0.727	0.467	0.451	0.365	0.45
BURM	0.446	**0**.**024**	0.328	0.39	0.293	0.222	0.402	0.492	0.392	0.331	0.277	0.262	0.281	0.394	0.259	0.229	**0**.**041**	0.501

The source populations were indicated in column and the aim populations were indicated in row, the diagonal values of the matrix were the probability with which individuals were assigned to their own reference populations, bold values were below 0.100.
