# Supplementary material for: Genetic diversity and population structure in Bactrocera correcta (Diptera: Tephritidae) inferred from mtDNA cox1 and microsatellite markers
Source: Sci Rep. 2016 Dec 8;6:38476. doi: 10.1038/srep38476 (PMC5144084; doi:10.1038/srep38476)
Supplement: Supplementary Information [file srep38476-s1.pdf]

Genetic diversity and population structure in *Bactrocera correcta* (Diptera: Tephritidae) inferred from mtDNA *cox1* and microsatellite markers

Yu-Jia Qin[1,§], Nopparat Buahom[1,2,§], Matthew N. Krosch[3], Yu Du[4], Yi Wu[5], Anna R Malacrida[6], Yu-Liang Deng[7], Jia-Qi Liu[8], Xiao-Long Jiang[4] and Zhi-Hong Li[1,*]

[1]College of Plant Protection, China Agricultural University, Beijing 100193, China;
[2]Office of Agriculture Regulation, Department of Agriculture, Ladyao, Chatuchak, Bangkok 10900, Thailand.
[3]School of Earth, Environmental and Biological Sciences, Queensland University of Technology, G.P.O. Box 2434, Brisbane 4000, QLD, Australia.
[4]Yunnan Entry-Exit Inspection and Quarantine Bureau, Kunming 650228, China.
[5]Academy of State Administration of Grain, Beijing 100037, China;
[6]Dipartimento di Biologia Animale, Università degli studi di Pavia, Piazza Botta, I27100 Pavia, Italy.
[7]Xishuangbanna Entry-Exit Inspection and Quarantine Bureau, Jinghong 666100, China.
[8]General Administration of Quality Supervision, Inspection and Quarantine of the People's Republic of China, Beijing 100088, China.

[§]These two authors (Yu-Jia Qin and Nopparat Buahom) contributed equally to this research.
Address: No. 2 Yuanmingyuan West Road, Haidian District, Beijing 100193, China.

[*]Corresponding author: lizh@cau.edu.cn (Zhi-Hong Li)

Table S1 Characterization of 12 microsatellite markers of *B. correcta*

**Locus**	***N***	***N*$_E$**	***I***	***H*$_O$**	***H*$_E$**	***Fis***	**PIC**
Bcor1	15	2.8137	1.4339	0.4968	0.6450	0.1068*	0.612
Bcor2	11	3.2737	1.4151	0.5931	0.6950	0.0957*	0.646
Bcor3	20	4.0562	1.7661	0.3446	0.7540	0.5277*	0.725
Bcor4	21	4.9162	1.9526	0.5212	0.7971	0.3042*	0.772
Bcor5	13	3.5342	1.4175	0.5419	0.7175	0.1830*	0.670
Bcor6	21	4.2824	1.8330	0.5631	0.7670	0.2544*	0.741
Bcor7	8	1.3776	0.5876	0.2670	0.2743	-0.0111	0.258
Bcor8	11	2.5975	1.3240	0.5610	0.6154	-0.0133	0.569
Bcor9	15	3.4835	1.5092	0.4968	0.7134	0.2907*	0.671
Bcor10	18	3.7198	1.8088	0.5308	0.7316	0.2516*	0.713
Bcor11	13	1.6979	0.7898	0.3864	0.4113	0.0019*	0.363
Bcor12	19	3.8287	1.7901	0.3691	0.7393	0.4730*	0.715

N: number of alleles; N_E: mean number of effective alleles; I: mean Shannon's information index; H_O: mean observed heterozygosity; H_E: mean expected heterozygosity; F_{IS}: within population inbreeding coefficient; PIC: polymorphic information content. *: deviation from HWE at $P<0.05$ after Bonferroni correction

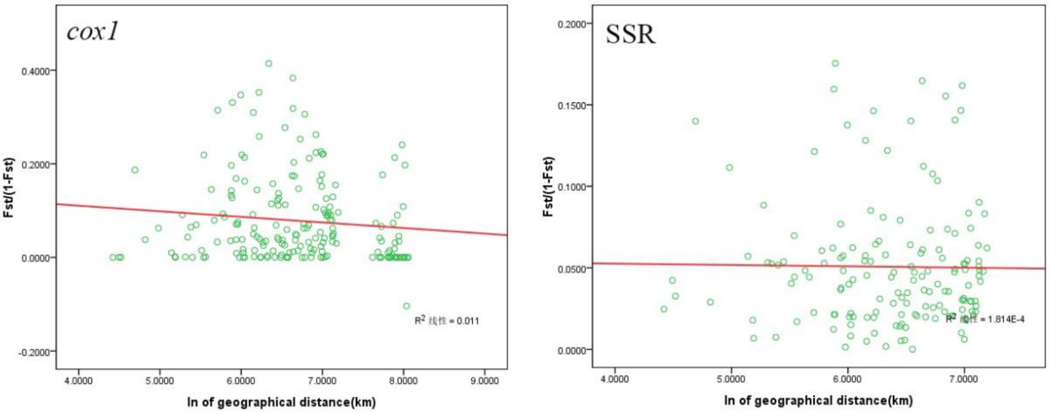

Figure S1. Mantel correlation tests of geographical and genetic distances based on two set of molecular makers of *B. correcta*

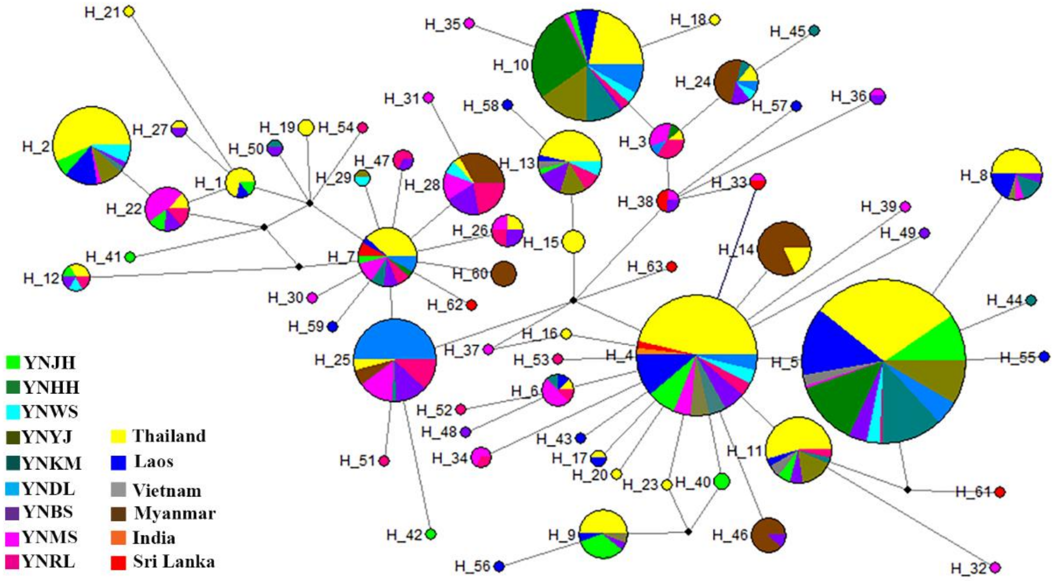

Figure S2. Median-Joining haplotype network of *B. correcta* based on mtDNA *cox1* data.

Note: Size of nodes and pie segments were proportional to haplotype frequency; small black circles represent median vectors (roughly equivalent to hypothetical unsampled haplotypes); length of the branched is proportional to number of mutational changes between haplotypes.

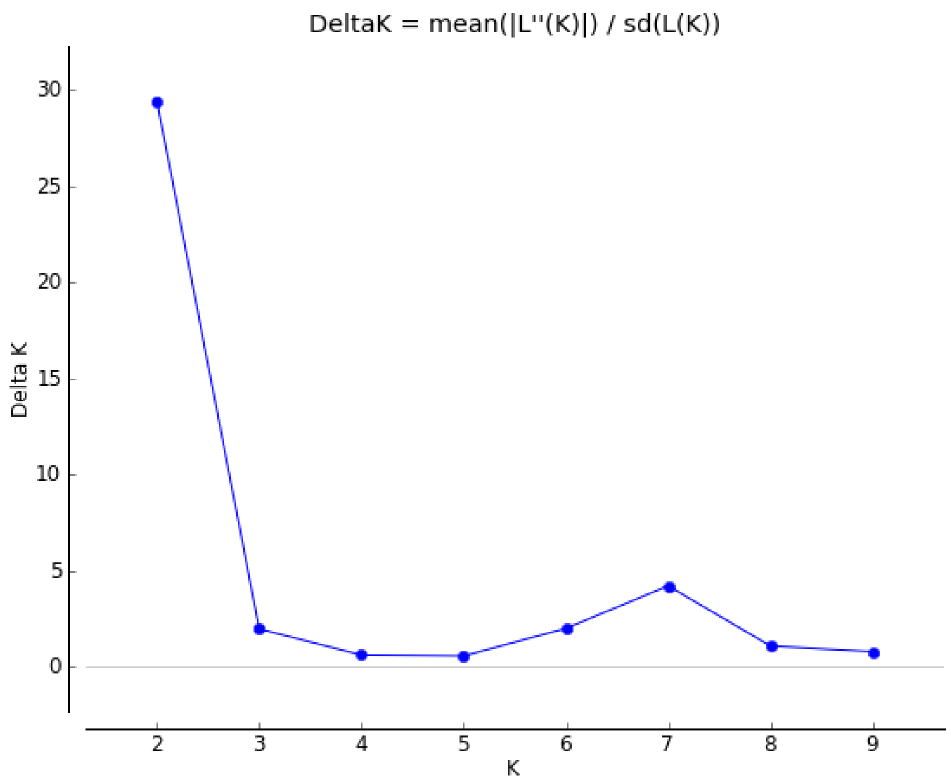

Figure S3. The graph of the best number of cluster of *B. correcta* from Structure Harvester

Table S2 SAMOVA results of *B. correcta* based on *cox1* data (K=2)

**Source of Variation**	**d. f.**	**Sum of squares**	**Variance components**	**Percentage of Variation**	**Fixation Indices**
Among groups	1	38.296	0.31330Va	12.28	F_{SC} = 0.06143
Among populations within groups	18	133.503	0.13743Vb	5.39	F_{ST} = 0.17673
Within populations	773	1623.062	2.09969Vc	82.33	F_{CT} = 0.12284
Total	792	1794.861	2.55042		

Table S3 AMOVA results of *B. correcta* based on *cox1* and microsatellite markers

	**Source of Variation**	**d. f.**	**Sum of squares**	**Variance components**	**Percentage of Variation**	**Fixation Indices**
cox 1	Among groups	1	38.917	0.31904Va	12.34	F_{SC} = 0.06112**
	Among populations within groups	18	134.782	0.13853Vb	5.36	F_{ST} = 0.17697**
	Within populations	773	1644.917	2.12796Vc	82.30	F_{CT} = 0.12339**
	Total	792	1818.615	2.58554		
**SSR**	Among groups	1	68.760	0.28152 Va	7.46	F_{IS}= 0.21817**
	Among populations within groups	16	229.773	0.11994 Vb	3.18	F_{SC}= 0.03434**
	Among individual within populations	763	3134.964	0.73587 Vc	19.50	F_{CT}= 0.07459**
	Within individuals	781	2059.500	2.63700 Vd	69.87	F_{IT}= 0.30133**
	Total	1561	5492.996	3.77433		

**P<0.001

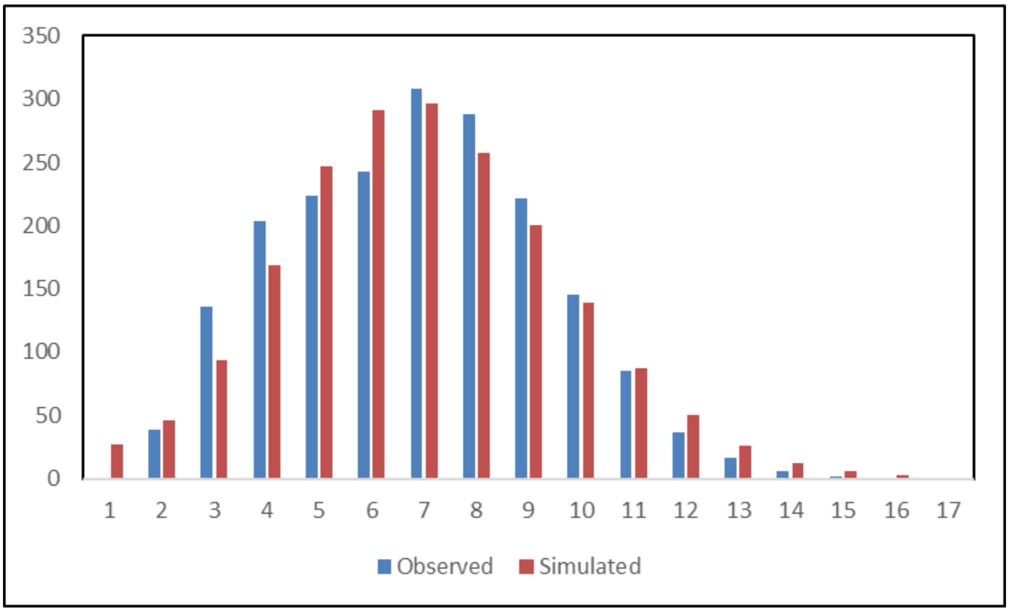

Figure S4. Mismatch distributions of the 793 *cox1* sequences of *B. correcta*
